# Sociodemographic and psychological parameters of adult’s commitment to exercise

**DOI:** 10.1192/j.eurpsy.2021.1232

**Published:** 2021-08-13

**Authors:** A. Zartaloudi, D. Christopoulos, M. Kelesi, O. Govina

**Affiliations:** 1 Nursing, University of West Attica, Athens, Greece; 2 Nursing, Psychiatric Hospital of Athens “Dafni”, Athens, Greece

**Keywords:** motivation, mental health, Physical Activity, athletics

## Abstract

**Introduction:**

Motivation is an important indicator of predicting an adult’s commitment to exercise so it is important to explore the reasons that may lead a person to participate in physical activity programs.

**Objectives:**

To investigate the socio-demographic and psychological parameters that motivate adults to participate in exercise programs and athletic activities.

**Methods:**

245 adults, being engaged in physical activity programs were given a questionnaire to collect information on socio-demographic characteristics, possible previous problems with body weight, type of exercise, frequency and main reason for their participation in exercise programs, as well as the somatometric characteristics of the participants.

**Results:**

It is noteworthy that participants’ motive for exercise was pleasure (for 46.1% of the participants), championship (for 20.8% of the participants), health reasons (for 18.4% of the participants), weight loss (for 7.8% of the participants) and improvement of physical appearance (for 6.9% of the participants). A greater percentage of male compared to female participants were engaged to exercise due to championship reasons, while more women than men exercised to a statistically significant extent in order to improve their appearance and for health reasons.

**Conclusions:**

Understanding the main factors that make individuals being engaged to physical activity may help health professionals to implement educational and counseling intervention programs regarding the positive effects of exercise on individuals’ mental and emotional health. Physical activity contributes to the improvement of their quality of life, which may be the most important issue for mental and public health.

